# An In Vitro Study of Chitosan-Coated Bovine Pericardium as a Dural Substitute Candidate

**DOI:** 10.3390/jfb14100488

**Published:** 2023-09-22

**Authors:** Asra Al Fauzi, Joandre Fauza, Heri Suroto, Muhammad Arifin Parenrengi, Wihasto Suryaningtyas, Prihartini Widiyanti, Nur Setiawan Suroto, Budi Utomo, Billy Dema Justia Wahid, Fitria Renata Bella, Yurituna Firda

**Affiliations:** 1 Department of Neurosurgery, Faculty of Medicine, Universitas Airlangga, Dr. Soetomo General Academic Hospital, Surabaya 60131, Indonesia; joandre.fauza.jf@gmail.com (J.F.); muhammad.arifin@fk.unair.ac.id (M.A.P.); wihasto-s@fk.unair.ac.id (W.S.); setiawan.dr@gmail.com (N.S.S.); dr.billywahid@gmail.com (B.D.J.W.); fitria.renata.bella-2014@fst.unair.ac.id (F.R.B.); 2 Department of Orthopedic and Traumatology, Faculty of Medicine, Universitas Airlangga, Dr. Soetomo General Academic Hospital, Surabaya 60131, Indonesia; heri.suroto@fk.unairac.id; 3 Biomedical Engineering Study Program, Department of Physic, Faculty of Science and Technology, Universitas Airlangga, Surabaya 60115, Indonesia; pwidiyanti@fst.unair.ac.id (P.W.); yurituna.firda-2021@fst.unair.ac.id (Y.F.); 4 Department of Public Health, Faculty of Medicine, Universitas Airlangga, Surabaya 60115, Indonesia; budiutom@gmail.com

**Keywords:** bovine pericardium, chitosan, dural substitute

## Abstract

Defects in the dura matter can be caused by head injury, and many cases require neurosurgeons to use artificial dura matter. Bovine pericardium is an option due to its abundant availability, adjustable size and characteristics, and because it has more collagen than porcine or equine pericardia. Nevertheless, the drawback of bovine pericardium is that it has a higher inflammatory effect than other synthetic dura matters. Chitosan has been shown to have a strong anti-inflammatory effect and has good tensile strength; thus, the idea was formulated to use chitosan as a coating for bovine pericardium. This study used decellularized bovine pericardial membranes with 0.5% sodium dodecyl sulphate and coatings containing chitosan at concentrations of 0.25%, 0.5%, 0.75%, and 1%. An FTIR test showed the presence of a C=N functional group as a bovine pericardium–chitosan bond. Morphological tests of the 0.25% and 0.5% chitosan concentrations showed standard pore sizes. The highest tensile strength percentage was shown by the membrane with a chitosan concentration of 1%. The highest degradation rate of the membrane was observed on the 7th and 14th days for 0.75% and 1% concentrations, and the lowest swelling ratio was observed for the 0.25% concentration. The highest level of cell viability was found for 0.75% chitosan. The bovine pericardium membrane with a 0.75% concentration chitosan coating was considered the optimal sample for use as artificial dura matter.

## 1. Introduction

A dura matter defect is complicated and may be caused by either a head injury or a clinical action, such as an intradural procedure for removing a brain tumor or a treatment for another pathology which requires an incision to be made in the dura matter [1]. Incisions in the dura matter can cause cerebrospinal fluid (CSF) leaks, which can be fatal [2]. The primary closure of the dura matter via sutures is an ideal method for preventing CSF leakage [3], but if the operation requires decompression or takes part of the dura matter, a replacement for the dura is needed. A good artificial dura matter must be acceptable to the body’s tissues (biocompatible), must not cause an inflammatory reaction and must be able to prevent CSF leakage and reactions to the surface of the brain tissue [4].

In the scope of biomaterials, bovine pericardium has potential because of its abundant availability and its size and characteristics. Bovine pericardial tissue contains collagen, which is the largest component of the extracellular matrix (ECM) and contributes to the elastic properties of the graft that allows it to conform to the host tissue. Several studies have shown that bovine pericardium has disadvantages such as a higher inflammatory effect compared to other synthetic dura matters [5].

In this research, chitosan was coated on the membrane. Chitosan is a natural polymer which has good biological properties, so it is often applied, especially in the medical and pharmaceutical fields. In the application of chitosan, the biomaterial has several advantages, namely being compatible in wound areas, good resistance to inflammatory processes, and being able to induce cell regeneration [6,7]. Chitosan has been proven to have a strong anti-inflammatory effect and has good tensile strength [8,9,10]. Due to the superior properties of the two polymers, the use of bovine pericardium with a chitosan coating is expected to provide new perspectives in the formulation of appropriate procedures and formulas to obtain artificial dura matter, assessments of which include an FTIR test, tensile test, morphological test, swelling test, degradation test, and cytotoxicity test. Consequently, this study aims to use bovine pericardium as the only control group as it represents a novel graft to be explored in the field of study. This study compared various concentrations of chitosan coatings to find the best effects with respect to its porosity, viability, tensile strength, swelling ratio, and degradation to fashion an enhanced artificial dural graft.

## 2. Materials and Methods

### 2.1. Materials

Bovine pericardium parietal sections, sodium dodecyl sulphate, glutaraldehyde, and medium-molecular-weight chitosan were obtained from Sigma-Aldrich, Palo Alto, CA, USA; 0.9% NaCl, acetic acid, NaOH, and phosphate-buffered saline were obtained from SAP Chemicals Bandung, Indonesia. Distilled water and BHK-21 cells were used for the cytotoxicity test.

### 2.2. Bovine Pericardium Tissue Preparation

Fresh parietal bovine pericardia from adult cattle were purchased from a local abattoir, placed in a cold ice box and transported to the regenerative medicine laboratory of stem cells and tissue banks at Dr. Soetomo General Academic Hospital. The tissues were rinsed with a 0.9% NaCl solution to remove blood and body fluids. Furthermore, decellularization was carried out using 0.5% sodium dodecyl sulphate (SDS), followed by fixation with 0.5% glutaraldehyde (GA) and washing using distilled water and a 0.9% NaCl solution to remove residue. After that, the sample was lyophilized in a freeze-dryer at temperatures between −13 °C and 50 °C for 48 h. A bovine pericardium without a chitosan coating was labeled as a control sample.

### 2.3. Bovine Pericardium Coating

Chitosan was dissolved in a 0.1 M acetic acid solution (pH = 3) at varying concentrations of 0.25%, 0.5%, 0.75%, and 1% (m/v) and stirred for 6 h or until homogeneous at room temperature. After that, the bovine pericardium samples were immersed in the chitosan solution for 6 h and dried at room temperature. All samples were rinsed with a 0.002 M NaOH solution until the pH of the surface became neutral, and they were then lyophilized again for 48 h. All the variations of the chitosan-coated bovine pericardium samples are listed in Table 1. Before the evaluation, all samples were sterilized with gamma irradiation; a dose of 25 kGy was applied in our study.

### 2.4. Morphological Analysis Using Scanning Electron Microscope (SEM)

Morphological tests were performed to observe the surface structure and pore diameter using SEM (Zeiss EVO MA 10, Jena, Germany). All samples were prepared by cutting them to a size of 1 × 1 cm, and then sputtered with palladium–gold. The samples were reviewed with a magnification of 500×. The pore diameter was measured with ImageJ software (Ver. System 7.0, Drexel University).

### 2.5. Fourier Transform Infrared (FTIR) Spectroscopy

FTIR spectroscopy characterization with an attenuated total reflectance (FTIR-ATR) method (Bruker-Alpha II, Billerica, MA, USA) was performed to determine the functional groups and the effect of chitosan coating on the molecular structure of the samples. The samples were cut to a size of 1 × 1 cm, and FTIR analysis was performed within the range of 4000–600 cm^−1^.

### 2.6. Tensile Strength Test

A tensile strength test was performed to determine the material’s resistance to loads; its mechanical characteristics were analyzed using an autograph universal testing machine. All samples were cut into dog bone shapes following the standard of the American Society for Testing Materials (ASTM) D638. Prior to testing, all samples were dipped in PBS to determine the biological surroundings of the dura matter.

### 2.7. Water Retention Test/Swelling Ratio

A water retention test was performed to observe the ability of the samples to absorb water, since this property is critical for the application of dural substitutes. All samples were cut to 1 × 1 cm, then they were weighed and labelled as W dry. After that, the samples were soaked in 10 mL of PBS solution for 4 h at a temperature of 37 °C. After that, the samples were placed on filter paper and then they were weighed and labelled as W wet. The percentage of water retention was obtained using the following formula:

$$\%Water\;retention=\frac{\left(W\;wet-W\;dry\right)}{W\;dry}\times 100\%$$

### 2.8. Degradation Test

A degradation test was performed to determine the ability of the samples to degrade due to interaction with static fluid. The in vitro degradation test was carried out by cutting all the samples measuring 1 × 1 cm and then the samples were weighed and registered as pre-test mass. All samples were immersed in 10 mL PBS solution at a temperature of 37 °C for 7 and 14 days. After that, the samples were placed in the dry oven for 24 h and then they were weighed and registered as post-degradation mass. The percentage of degradation were obtained using the following formula:

$$\%Degradation=\frac{post\;degradation\;mass-pre\;degradation\;mass}{pre\;degradation\;mass}\times 100\%$$

### 2.9. Cytotoxicity Test

The cytotoxicity test is used to see the non-toxicity of the sample. This test aims to determine the percentage of living cells when the sample is exposed to the cells. Baby hamster kidney (BHK-21) cells, which are easy to culture, were used in this test.

### 2.10. Statistical Analysis

All data results were presented using the mean value. Statistical analysis was performed using IBM SPSS Statistics Version 21.0 (IBM Technology Lifecycle Sevices, Armonk, NY, USA) to evaluate significant differences between the groups. The data were considered significant when the *p* value was <0.05.

## 3. Results

### 3.1. Morphological Testing Using SEM

**Figure 1:**
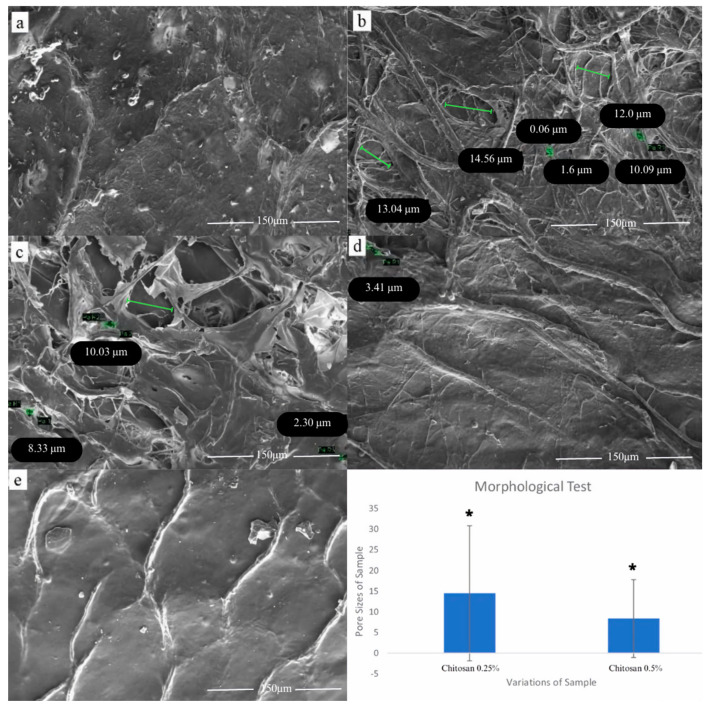
The morphological structure of the sample: (**a**) bovine pericardium; (**b**) bovine pericardium coated with chitosan 0.25%; (**c**) chitosan 0.5%; (**d**) chitosan 0.75%; (**e**) chitosan 1%. Statistical analysis of chitosan: 0.25% and 0.5% * *p* < 0.05.

Morphological testing was carried out on all variations of the sample, including control samples and variations of 0.25%, 0.5%, 0.75%, and 1% chitosan coating. Based on Figure 1, no pores were found in the control samples, with bovine pericardium–chitosan mixtures of 0.75% and 1%. Meanwhile, the 0.25% and 0.5% bovine pericardium–chitosan samples had average pore sizes of 14.51 ± 16.36 µm and 8.38 ± 9.44 µm. Statistical analysis of the variations in the treatment of chitosan-coated bovine pericardium with concentrations of 0.25% and 0.5% was performed, finding that the data were normally distributed and were homogeneous with *p* > 0.053. Tests using the independent sample test showed that variations in the treatments with the addition of chitosan had variable impacts, with a significance value of (2-tailed) *p* = 0.015 < 0.05 (shown in Figure 1).

### 3.2. FTIR Spectroscopy

Functional group testing was carried out to determine the content of the chemical components of each sample. Spectrum FTIR for the control bovine pericardium sample showed at 3305.30 cm^−1^, which indicates the presence of type I collagen. The wavelengths of 2920.66 cm^−1^ and 2853.28 cm^−1^ indicate CH stretching and a wavelength of 1636.34 cm^−1^ indicates NH bends [11].

**Figure 2:**
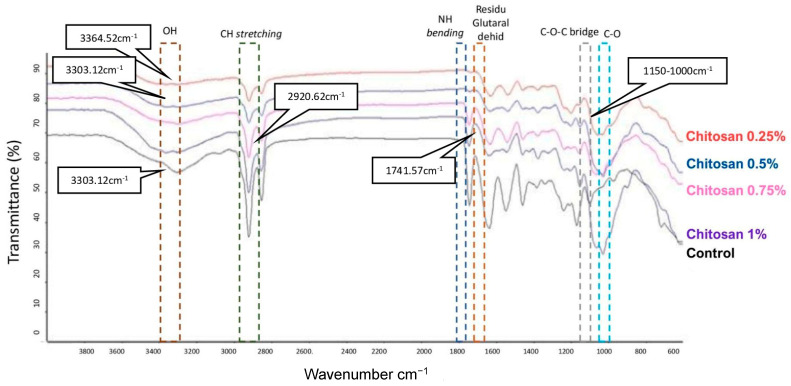
Results of FTIR spectra on chitosan-coated bovine pericardial membrane with various concentrations of 0% (control); chitosan 0.25%; chitosan 0.5%; chitosan 0.75%; chitosan 1%.

In the various coatings of chitosan with concentrations of 0.25%, 0.5%, 0.75%, and 1%, there were functional groups OH, CH stretching, and NH bending successively, including at a concentration of 0.25% chitosan, which showed at 3364.52 cm^−1^, 2920.06 cm^−1^, and 1628.19 cm^−1^. At 0.5% chitosan, the absorption peaks were at 3303.12 cm^−1^, 2919.72 cm^−1^, and 1633.53 cm^−1^. Chitosan variations of 0.75% are found at wavelengths of 3296.32 cm^−1^, 2920.62 cm^−1^, and 1631.60 cm^−1^, while the 1% coating was found at 3363.88 cm^−1^, 2912.18 cm^−1^, and 1635.70 cm^−1^. This evidenced that the functional group absorption areas which are typical of bovine were present in all variations. The functional group absorption areas which are typical of chitosan were indicated by the presence of a COC bridge and CO at absorption waves around 1150 cm^−1^ and 1000 cm^−1^ in treated samples or in those coated with chitosan. Chitosan coating with concentrations of 0.25% and 0.5% resulted in inactivation (masking) of glutaraldehyde (C=O) residues, which was indicated by the disappearance of the sharpness of the wave absorption [12] and a lower wavenumber shift (1741.57; 1735.22; 1738.54). This indicated the presence of hydrogen bonds between the molecules of the two materials [13] (shown in Figure 2).

### 3.3. Tensile Strength and Elongation

The ultimate tensile strength (UTS) value is the maximum stress a material can achieve before it undergoes plastic deformation. When the sample is given a tensile force, deformation or elongation will occur. UTS analysis is considered as a parameter that aims to determine the toughness or strength of a material [14]. Mechanical testing of a material is one of the required methods for evaluating implants in the body. Table 2 shows variations in the control and in the addition of chitosan at 0.25%, 0.5%, 0.75%, and 1% were (22.302 ± 3.578) MPa, (2.854 ± 0.907) MPa, (2.726 ± 0.879) MPa, (2.818 ± 1.088) MPa, and (1.733 ± 1.070) MPa, respectively. The results of the tensile value measurements showed that all the variations of the chitosan concentration showed the tensile value standards for dura matter applications. The standard tensile value is in the range of 0.6–16 Mpa [14]. The elongation calculation values for each sample are (60.283 ± 15.411)%, (20.312 ± 4.101)%, (19.696 ± 3.546)%, (22.158 ± 4.912)%, and (11.980 ± 4.556)%, respectively.

**Figure 3:**
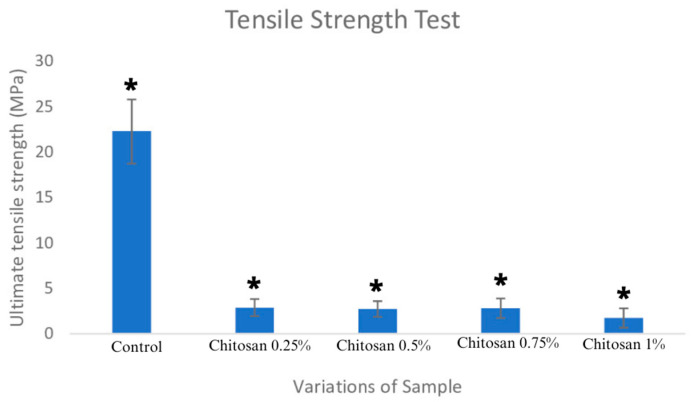
Tensile strength test; * *p* < 0.05.

The data were normally distributed and were homogeneous. This was supported by a significance value of *p* = 0.24 > 0.05. A one-way ANOVA test with a significance value of *p* = 0.000 < 0.05 showed that the variation in the addition of chitosan for each sample had varying impacts. Statistical analysis showed that the data were normally distributed and were homogeneous. This was supported by a significance value of *p* = 0.69 > 0.05. A one-way ANOVA test with a significance value of *p* = 0.000 < 0.05 showed that the variation in the addition of chitosan for each sample had varying impacts. The Tukey post hoc test showed that the control sample had a different impact than the other four samples (showed in Figure 3).

### 3.4. Water Retention Test/Swelling Test

**Figure 4:**
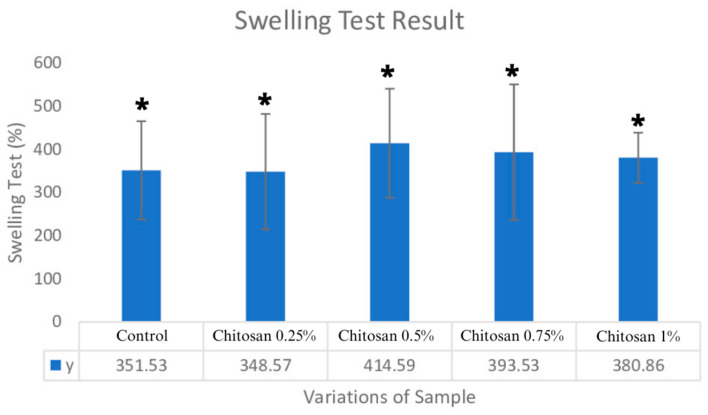
Swelling test result; * *p* > 0.05.

The swelling ratio based on the result showed that the 0.5% variation has the highest value among the other variations. The ratio value was (414.59 ± 126.42)%, which indicates that the sample was more hydrophilic; this is aligned with chitosan’s characteristics [15]. In the bovine pericardium sample, the study’s control sample, the swelling ratio was (351.53 ± 113.88)%. With the addition of chitosan compounds, it is hoped that there will be no significant changes in the nature or shape of the bovine pericardium grafts. Based on experimental results, the swelling ratio was (348.57 ± 133.94)% with the addition of 0.25% chitosan concentration. There was a reduction of ±0.84% swelling compared with the control. Based on experimental results, the swelling ratio was (348.57 ± 133.94)% at the addition of 0.25% chitosan concentration. There was a reduction of ±0.84% swelling compared with the control. Based on statistical analysis (showed in Figure 4), the data were homogeneous and normally distributed. This was supported by a significance value of *p* = 0.715. Further testing using one-way ANOVA showed that there was a significant difference for each variation with a significance value of *p* = 0.286.

### 3.5. Degradation

**Figure 5:**
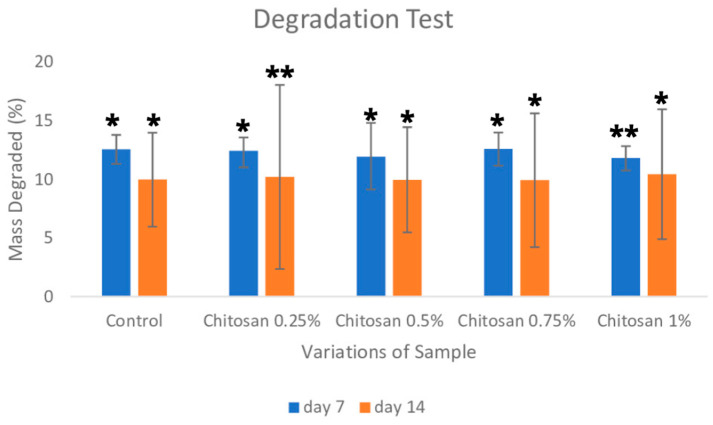
Degradation test result; * *p* < 0.05 and ** *p* > 0.05.

This test used phosphate-buffered saline which stimulates fluids in the body, and the test was carried out in an incubator at 37 °C. The degradation test, with a duration of 14 days, mirrors the longest inflammatory phase that occurs in response to tissue damage/dura matter defects. The inflammatory phase is followed by the proliferative phase, which triggers fibroblasts and tissue remodelling [16,17]. In the bovine study with chitosan at a concentration of 0.75%, the average presentation was 12.60% on the 7th day and 9.931% on the 14th day (showed in Figure 5).

The statistical analysis test showed that the data were normally distributed and homogeneous, with a significance value of *p* = 0.387 > α. In the degradation sample test, there were two variations, namely variations in the treatment of control samples coated with chitosan concentrations of 0.25%, 0.5%, 0.75%, and 1%. The next variation was the time on the 7th and 14th day. For statistical analysis, we used a factorial test with a non-variating general model. This analysis showed the relationship between variations in the treatments and variations in the day through the percentage of degraded mass, with a significance value of *p* = 0.508. Furthermore, the one-way ANOVA test showed a *p* value of 0.000. The next analysis was Tukey’s post hoc text which showed a more optimal mass change in the sample with 0.25% chitosan coating. Based on statistical analysis, it showed that the variation in treatment has a significant effect on mass changes.

### 3.6. Cytotoxicity

The results showed that the samples with the addition of chitosan concentrations of 0.25% and 0.75% were non-toxic. However, the results showed no diversity due to their non-viability at the addition of 0.5% and 1% chitosan concentrations. Viability values in the cell viability results from the bovine–chitosan pericardium cytotoxicity test, with concentrations of 0.25% and 0.75%, produced results of (94.67 ± 6.57)% and (96.76 ± 0.06)%, respectively; these were higher than those obtained for the bovine pericardium preserved with glycerol (50%).

**Figure 6:**
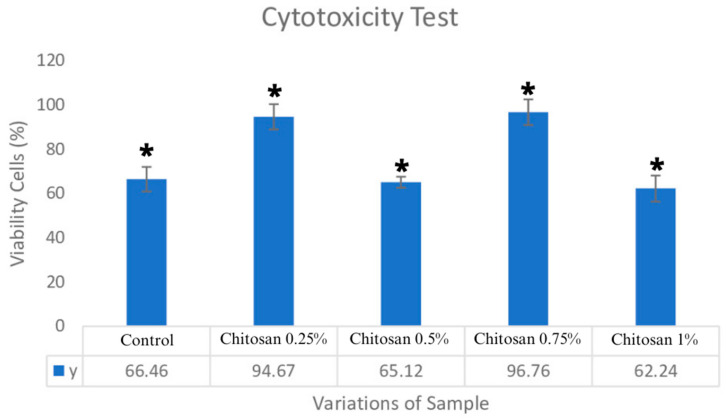
Cytotoxicity test results; * *p* < 0.05.

As shown in Figure 6, statistical analysis showed that all data were normally distributed and were homogeneous. This is evidenced by the significance value of *p* < 0.099. The one-way ANOVA test showed that there was a difference in meaning with the addition of chitosan coating to the bovine pericardium control sample. Tukey’s HSD follow-up test showed that the control sample had a significant difference in chitosan coating, with concentrations of 0.25% and 0.75%, but no significant difference with a 1% chitosan concentration. Based on the statistical analysis table, the best variation of chitosan is found at a concentration of 0.75%, with cell viability results of (95.76 ± 5.78)%.

## 4. Discussion

Chitosan-coated bovine pericardium has been successfully synthesized using freeze-drying method. The samples used were the control sample and those with the addition of chitosan coatings at 0.25%, 0.5%, 0.75%, and 1% concentration. The samples were characterized using an FTIR test, a morphological test, a tensile test, a degradation test, a cytotoxicity test, and swelling test.

Since this study is currently in the preliminary in vitro stage, we do not have cell cultures; therefore, we relied solely on SEM to examine the physical characteristics of the samples, specifically their pore availability and each size. Morphological testing showed that no pores were found in the bovine pericardium control samples; this finding is reflective of its morphology, in that the bovine pericardium tends to comprise more flat-surfaced cells, containing very few microvilli. Microvilli on the pericardial surface serve as friction-bearing structures and as a means of increasing the surface area available for fluid and solute transport, providing a permeability seal [18]. The thickness of the bovine pericardium varies greatly from 984 ± 147 µm to 681 ± 141 µm in very close proximity. The normal bovine parietal pericardium varies in overall thickness from 0.3 to 0.7 mm and is composed of three layers consisting of the serous, fibrous, and epipericardial connective tissue layers [19]. Differences in the thickness of the pericardium and the type of wave variation in the collagen fibril bundles can lead to unequal biomolecular proportions in the samples, which results in no pores being found at chitosan concentrations of 0.75% and 1%.

Pore size is an important factor in tissue engineering, when the aim is to mimic the structure of the extracellular matrix. This is because it can be used to increase biocompatibility, migration activity, diffusion, and cell attachment to samples which supports tissue regeneration [8,20,21]. Pore size affects the permeability of the scaffold; higher pore sizes increase the permeability of the scaffold. High permeability causes CSF (cerebrospinal fluid) to leak, causing complications after duraplasty surgery. Using the appropriate scaffold prevents CSF leakage [22].

The elongation results for all variations in chitosan concentration showed the presence of chitosan in a standard range of 7–20% [23]. A high Young’s modulus value indicates that the material is difficult to stretch elastically; meanwhile, a low value indicates that the material can be flexed elastically [24]. Based on these calculations, the addition of chitosan can increase the Young’s modulus value. Chitosan coatings on bovine samples were able to increase the density of the solution due to the addition of polymer compounds [25]. In this study of the coating of bovine pericardium with chitosan, we obtained a higher average yield of tensile strength and elongation presentation of bovine pericardium than that obtained in the study of Stieglmeier et al., 2021 [19]. The selection of a biomechanical material has elasticity and thickness characteristics most similar to those of dura matter tissue will have a positive effect in reducing pressure during tissue sutures and surgery [14]. Thus, such a development would prevent complications during operations.

The result of the swelling ratio shows that none of the increased concentrations of chitosan attracted hydrogen, which would have made it absorb more liquid [13]. Differences in the thickness of the pericardium and in the type of collagen fibril bundle wave variation can lead to an unequal proportion of biomaterials in the sample, which causes a decrease in the presentation of swelling ratios of [4] 0.75% and 1%. At a concentration of 0.5%, there was an increase in the swelling ratio of (414.59 ± 126.42)%. There was a percentage increase of ±18.93% in the control sample. Then, it decreased more with the addition of chitosan at concentrations of 0.75% and 1%. The swelling test results from the control and pericardium–bovine membrane concentrations of 0.25%, 0.5%, 0.75%, and 1% were found to be much lower than those from the conventional dural grafts of 850 ± 65%.

In the study by Deng et al. (2021), tests were carried out on the characterization of three types of samples: CS/BS samples (high-molecular-weight chitosan–bacterial cellulose); O-CMCH/BC (ocarboxymethyl chitin–bacterial cellulose); and BiCA dura matter substitution with BiGA (two layers of composite polysaccharide scaffold cross-linked with citric acid) and BiGA (two-layer composite polysaccharide scaffold cross-linked with glutaraldehyde). The dural substitution results from BiCA showed swelling test results of (694 ± 38)%, and from BiGA these results were (787 ± 72)% [19]; these results were higher than those for the swelling test at the highest yield of the bovine pericardium–chitosan concentration 0.5% with a swelling ratio (414.59 ± 126.42)% [26].

The temperature factor is something that can affect the ability of the sample to swell. Previous research has shown that the swelling ability at a low temperature of 10 °C shows a lower ratio than that at a high temperature of 50 °C. High temperatures can weaken intermolecular bonds, resulting in an increased swelling ratio value [27]. This led to the testing process being carried out at a temperature of 37 °C, which is a simulation of human body temperature. The greater the swelling presentation is, the smaller the pore size will be. This is caused by increased liquid in the membrane, thus having a compression effect on the pores on the surface of the membrane [13,15]. The nature of the high swelling presentation can provide a compression effect on the surrounding tissue [28]. Excessive compression can cause tearing or rupture of the artificial dura matter, which causes CSF leakage. Many cases of leaking CSF progress to meningitis [29]. Another complication that often occurs after duraplasty is that leaking CSF accumulates and forms a pseudo meningocele [30].

The ability of biomaterials to control the degradation process is very important; this is because, if the degradation rate is too slow, it can disrupt the tissue repair process, whereas if the degradation rate is too fast then new tissue will not form [31]. The addition of glycerol can increase the free volume between the cellulose polymer chains and increase the free hydroxyl groups, which will form hydrogen bonds with liquids, thereby increasing the ability of the membrane to absorb liquids. Glycerol can reduce interactions between biopolymer molecules and can increase degradation; this is because it is hydrophilic, so the affinity of polymer molecules is higher, enabling the attraction of solutions [32]. When the degradation process occurs too early, it causes a decrease in the mechanical properties of the biomaterial and causes postoperative failure [31]. When degradation of the dura matter graft is too fast, it can create an open defect in the dura. Postoperative complications include CSF leakage and a risk of immediate infection which can cause wound dehiscence and subgaleal fluid collection.

Mitochondria are a dynamic cellular organelles which participate in cellular homeostasis. It has been observed that energy production differs depending on cell type. The metabolism of BHK-21 cells shows that they comprise an adherent cell line, so they are often used in regenerative medicine [31]. BHK-21 cells grow in an adherent manner; they comprise an anchor-dependent mammalian cell line with a fibroblast growth pattern in standard cell culture conditions which is similar to others, including fetal bovine serum [33]. The cytotoxicity test results showed that all the variant samples were non-toxic. An addition of 0.75% chitosan had a result of (96.76 ± 0.06)%, which is the optimal result for viable cells. A previous study proved that chitosan can increase cell viability. The addition of chitosan makes the scaffold dura matter more hydrophilic; it could be possible that the cells adhered to the scaffold dura matter [16]. A cytotoxicity test can be used to evaluate materials; the test is based on the host response mechanism during the process of implanting biomaterial. The host response process includes three phases, namely the inflammatory phase, the proliferation phase, and the tissue regeneration phase.

Previous studies have shown that chitosan can reduce inflammatory effects due to the number of cytokines present at the start of the inflammatory process and the amounts of TNF-α and IL-6, which are pro-inflammatory cytokines that are released early in the inflammatory process. In addition, chitosan has also been shown to reduce the rate of the release of nitric oxide, as induced by lipopolysaccharide in inflammatory responses [34,35]. Specific tests for pro-inflammatory cytokines that potentially enhanced with the chitosan-coated graft were not performed. This was because the main purpose of this study was to qualitatively determine, over a value of 70%, whether the standard artificial dural graft was viable and to determine which ratio of chitosan was the most effective. Cells for the two variations were determined to be (94.67 ± 6.57)% and (95.76 ± 2.36)%. At chitosan concentrations of 0.5% and 1%, the cell viability results were 65.12% and 62.24%; the results were lower than those of the control (66.46%). According to previous research, the addition of chitosan can also reduce cell viability values. This is due to the presence of acetic acid residue, which is used as a solvent for chitosan [24]. Compounds that can induce cell death include acetic acid, sodium bicarbonate, potassium carbonate, lithium acetate, and formic acid [36]. In addition, differences in the thickness of the pericardium and the type of collagen fibril bundle wave variation can create an unequal proportion of biomolecules in the sample, which results in a decrease in the viability of the chitosan concentrations of 0.5% and 1%. Normal bovine parietal pericardium varies in its overall thickness from 0.3 to 0.7 mm and consists of three layers consisting of serous, fibrous, and epipericardial connective tissue layers. The serosa tissue is formed by a surface layer of thin, flattened mesothelial cells facing the pericardial cavity and by narrow sub-mesothelial spaces. The fibrous tissue contains flat, overlapping collagen bundles that have a wavy appearance and are oriented in many directions [18]. Further in vivo studies are required to investigate bovine pericardium–chitosan graft impact on cytokines.

## 5. Conclusions

The physical characteristics of the chitosan pericardial membrane for each chitosan concentration, as obtained from the FTIR functional group test, indicated the presence of a bovine pericardium–chitosan synthesis bond. The morphological test results of chitosan concentrations of 0.25% (6.843–10.09 µm) and 0.5% (8.191–27.47 µm), with bovine pericardium, showed pore sizes that were in accordance with the required standards. Increases in chitosan concentration led to a decrease in ultimate tensile strength (UTS) values and a decrease in sample elongation, where the average values of UTS and elongation met the standard values of artificial dura matter. The highest UTS value for the bovine pericardial membrane with chitosan was obtained at a concentration of 0.25% (2.854 ± 0.907) MPa, and the elongation value was (22.158 ± 4.912)%. The lowest swelling ratio of chitosan at a concentration of 0.25% was (348.57 ± 133.94)% and at 0.5% the ratio was (414.59 ± 126.42)%. On the 7th and 14th days, the highest percentage of degradation of the chitosan–bovine pericardial membrane was found in the samples with chitosan concentrations of 0.75% and 1%. The cytotoxicity of the sample with a 0.75% chitosan concentration was (95.76 ± 5.78)%. The sample with a bovine pericardial membrane with a 0.75% concentration chitosan coating was determined to be the best sample through the characterization tests.

## Figures and Tables

**Table 1:** Variations of chitosan coatings.

Sample	Concentration of Chitosan (*w/v*)%
A (BP without chitosan coating)	0
B (BP, with chitosan coating)	0.25
C (BP, with chitosan coating)	0.5
D (BP, with chitosan coating)	0.75
E (BP, with chitosan coating)	1

**Table 2:** Compressive strength test results.

Sample	A (mm^2^)	F_max_ (N)	Elongation/Strains (%)	UTS (MPa)
Control (bovine pericardium)	2.42	53.559419	60.28290289	22.30212767
Sample A (bovine pericardium + chitosan 0.25%)	1.918	5.59415514	20.31215049	2.8540068
Sample B (bovine pericardium + chitosan 0.5%)	1.0272	5.265274	19.69608871	2.7258298
Sample C (bovine pericardium + chitosan 0.75%)	1.04	3.687771	22.15770568	2.8183967
Sample D (bovine pericardium + chitosan 1%)	1.1656	1.896028	11.98032766	1.7334682

## Data Availability

The processed data required to reproduce these findings cannot be shared at this time as the data also form part of an ongoing research.

## References

[1] Ginsberg L. (2005). Lecturer Note Neurology 8th Edition. J. Neurol. Neurosurg. Psychiatry.

[2] Hobbs C.G.L., Darr A., Carlin W.V. (2011). Management of intra-operative cerebrospinal fluid leak following endoscopic trans-sphenoidal pituitary surgery. J. Laryngol. Otol..

[3] Protasoni M., Sangiorgi S., Cividini A., Culuvaris G.T., Tomei G., Dell’Orbo C., Raspanti M., Balbi S., Reguzzoni M. (2011). The collagenic architecture of human dura mater. J. Neurosurg..

[4] Danish S.F., Samdani A., Hanna A., Storm P., Sutton L. (2006). Experience with acellular human dura and bovine collagen matrix for duraplasty after posterior fossa decompression for Chiari malformations. J. Neurosurg..

[5] MacEwan M.R., Kovacs T., Osbun J., Ray W.Z. (2018). Comparative analysis of a fully-synthetic nanofabricated dura substitute and bovine collagen dura substitute in a large animal model of dural repair. Interdiscip. Neurosurg. Adv. Tech. Case Manag..

[6] Kranokpiraksa P., Pavcnik D., Kakizawa H., Uchida B.T., Jeromel M., Keller F.S., Rösch J. (2010). Hemostatic efficacy of chitosan-based bandage for closure of percutaneous arterial access sites: An experimental study in heparinized sheep model. Radiol. Oncol..

[7] Sandoval-Sánchez J.H., Ramos-Zúñiga R., Luquín De Anda S., López-Dellamary F., Gonzalez-Castañeda R., Ramírez-Jaimes J.D.L.C., Jorge-Espinoza G. (2012). A new bilayer chitosan scaffolding as a dural substitute: Experimental evaluation. World Neurosurg..

[8] Angtika R.S., Widiyanti P., Aminatun A. (2018). Bacterial cellulose-chitosan-glycerol biocomposite as artificial dura mater candidates for head trauma. J. Biomim. Biomater. Biomed. Eng..

[9] Pogorielov M., Kravtsova A., Reilly G.C., Deineka V., Tetteh G., Kalinkevich O., Pogorielova O., Moskalenko R., Tkach G. (2017). Experimental evaluation of new chitin–chitosan graft for duraplasty. J. Mater. Sci. Mater. Med..

[10] Widiyanti P., Jabbar H., Rudyardjo D.I. (2017). Effects of variation of chitosan concentration on the characteristics of membrane cellulose bacteria-chitosan biocomposites as candidates for artificial dura mater. AIP Conf. Proc..

[11] Santos M.H., Silva R.M., Dumont V.C., Neves J.S., Mansur H.S., Heneine L.G.D. (2013). Extraction and characterization of highly purified collagen from bovine pericardium for potential bioengineering applications. Mater. Sci. Eng. C.

[12] Gallyamov M.O., Chaschin I.S., Khokhlova M.A., Grigorev T.E., Bakuleva N.P., Lyutova I.G., Kondratenko J.E., Badun G.A., Chernysheva M.G., Khokhlov A.R. (2014). Collagen tissue treated with chitosan solutions in carbonic acid for improved biological prosthetic heart valves. Mater. Sci. Eng. C.

[13] Nie B., Stutzman J., Xie A. (2005). A vibrational spectral maker for probing the hydrogen-bonding status of protonated Asp and Glu residues. Biophys. J..

[14] Agrawal P., Pramanik K. (2017). Fabrication of chitosan-based nanofibrous scaffold using free surface electrospinning for tissue engineering application. Bioteknologi.

[15] Liao C.J., Wang W.H., Liang H.C., Su Y.C., Hsu P.C., Wang Y.M., Tsai Y.-H., Chen Y., Tseng S.-H. (2014). A novel foamy collagen as a dural substitute. Biomed. Eng.-Appl. Basis Commun..

[16] Suroto N.S., Al Fauzi A., Widiyanti P., Bella F.R. (2023). Biocompatibility Evaluation of Electrospun Poly-L lactic Acid-Chitosan Immobilizied with Heparin as Scaffold for Vascular Tissue Repair. J. Sci. Adv. Mater. Devices.

[17] Li J., Chen J., Kirsner R. (2007). Pathophysiology of acute wound healing. Clin. Dermatol..

[18] Ishihara T., Ferrans V.J., Jones M., Boyce S.W., Roberts W.C. (1981). Structure of bovine parietal pericardium and of unimplanted Ionescu-Shiley pericardial valvular bioprostheses. J. Thorac. Cardiovasc. Surg..

[19] Stieglmeier F., Grab M., König F., Büch J., Hagl C., Thierfelder N. (2021). Mapping of bovine pericardium to enable a standardized acquirement of material for medical implants. J. Mech. Behav. Biomed. Mater..

[20] Bružauskaitė I., Bironaitė D., Bagdonas E., Bernotienė E. (2016). Scaffolds and cells for tissue regeneration: Different scaffold pore sizes—Different cell effects. Cytotechnology.

[21] Fiqrianti I.A., Widiyanti P., Cahyani N.R., Bella F.R. (2018). Poly-L-lactic Acid (PLLA)-Chitosan-Collagen Electrospun Tube for Vascular Graft Application. J. Funct. Biomater..

[22] Wei J., Qian H., Liu Y., Liu J., Zhao R., Yang X., Zhu X., Chen R., Zhang X. (2018). Application of osteoinductive calcium phosphate ceramics in children’s endoscopic neurosurgery: Report of five cases. Regen. Biomater..

[23] Zhuravlova I.P., Vovk Y.N. (2012). Biomechanical properties of cerebral falx of human dura mater in adult human. Surg. Donnbass.

[24] Kizmazoglu C., Aydin H.E., Kaya I., Atar M., Husemoglu B., Kalemci O., Sozer G., Havitcioglu H. (2019). Comparison of biomechanical properties of dura mater substitutes and cranial human dura mater: An in vitro study. J. Korean Neurosurg. Soc..

[25] Thomas M.S., Pillai P.K.S., Faria M., Cordeiro N., Barud H., Thomas S., Pothen L.A. (2018). Electrospun polylactic acid-chitosan composite: A bio-based alternative for inorganic composites for advanced application. J. Mater. Sci. Mater. Med..

[26] Deng W., Tan Y., Riaz Rajoka M.S., Xue Q., Zhao L., Wu Y. (2021). A new type of bilayer dural substitute candidate made up of modified chitin and bacterial cellulose. Carbohydr. Polym..

[27] Amaral J.B.C.G., Ratnasari D., Trisanti P.N., Sumarno S., Ningrum E.O. (2016). Pengaruh Perubahan Suhu Pada Properti Adsorpsi Dan Desorpsi Thermosensitive NIPAM-Co-DMAAPS Gel. Seminar Nasional Teknik Kimia “Kejuangan”.

[28] Johansson B., Alderborn G. (2001). The effect of shape and porosity on the compression behaviour and tablet forming ability of granular materials formed from microcrystalline cellulose. Eur. J. Pharm. Biopharm..

[29] Horowitz G., Fliss D.M., Margalit N., Wasserzug O., Gil Z. (2011). Association between cerebrospinal fluid leak and meningitis after skull base surgery. Otolaryngol.-Head Neck Surg..

[30] Couture D., Branch C.L. (2003). Spinal pseudomeningoceles and cerebrospinal fluid fistulas. Neurosurg. Focus.

[31] Burke G., Barron V., Geever T., Geever L., Devine D.M., Higginbotham C.L. (2019). Evaluation of the materials properties, stability and cell response of a range of PEGDMA hydrogels for tissue engineering applications. J. Mech. Behav. Biomed. Mater..

[32] Popvic A., Drljaca J., Popovic M. (2022). Mitochondrial energy metabolism in baby hamster kidney (BHK-21/C13) Cells treated with karnozin EXTRA. Int. J. Morphol..

[33] Veronika D., Florian P., Aline Z., Beer M., Eschbaumer M. (2021). Adherent and suspension baby hamster kidney cells have a different cytoskeleton and surface receptor repertoire. PLoS ONE.

[34] Ghasemlou M., Khodaiyan F., Oromiehie A. (2011). Physical, mechanical, barrier, and thermal properties of polyol-plasticized biodegradable edible film made from kefiran. Carbohydr. Polym..

[35] Azuma K., Osaki T., Minami S., Okamoto Y. (2015). Anticancer and Anti-Inflammatory Properties of Chitin and Chitosan Oligosaccharides. J. Funct. Biomater..

[36] Lastauskiene E., Zinkevičiene A., Girkontaite I., Kaunietis A., Kvedariene V. (2014). Formic acid and acetic acid induce a programmed cell death in pathogenic Candida species. Curr. Microbiol..

